# Supplementary material for: A Zn(II)-Based Sql Type 2D Coordination Polymer as a Highly Sensitive and Selective Turn-On Fluorescent Probe for Al3+
Source: Molecules. 2021 Dec 6;26(23):7392. doi: 10.3390/molecules26237392 (PMC8658932; doi:10.3390/molecules26237392)
Supplement: Supplementary file 1 [file molecules-26-07392-s001.zip › Supplementary Materials.pdf]

Supplementary Materials

A Zn(II)-based sql type 2D coordination polymer as a highly sensitive and selective turn-on fluorescent probe for Al^{3+}

Dmitry I. Pavlov [1], Alexey A. Ryadun[1] and Andrei S. Potapov [1,*]

[1] Nikolaev Institute of Inorganic Chemistry, Siberian Branch of the Russian Academy of Sciences, 3 Lavrentiev Ave., 630090 Novosibirsk, Russia; pavlov@niic.nsc.ru (D.I.P.), potapov@niic.nsc.ru (A.S.P.), ryadun@niic.nsc.ru (A.A.R.)

* Correspondence: potapov@niic.nsc.ru; Tel.: +7 (383) 330-94-90

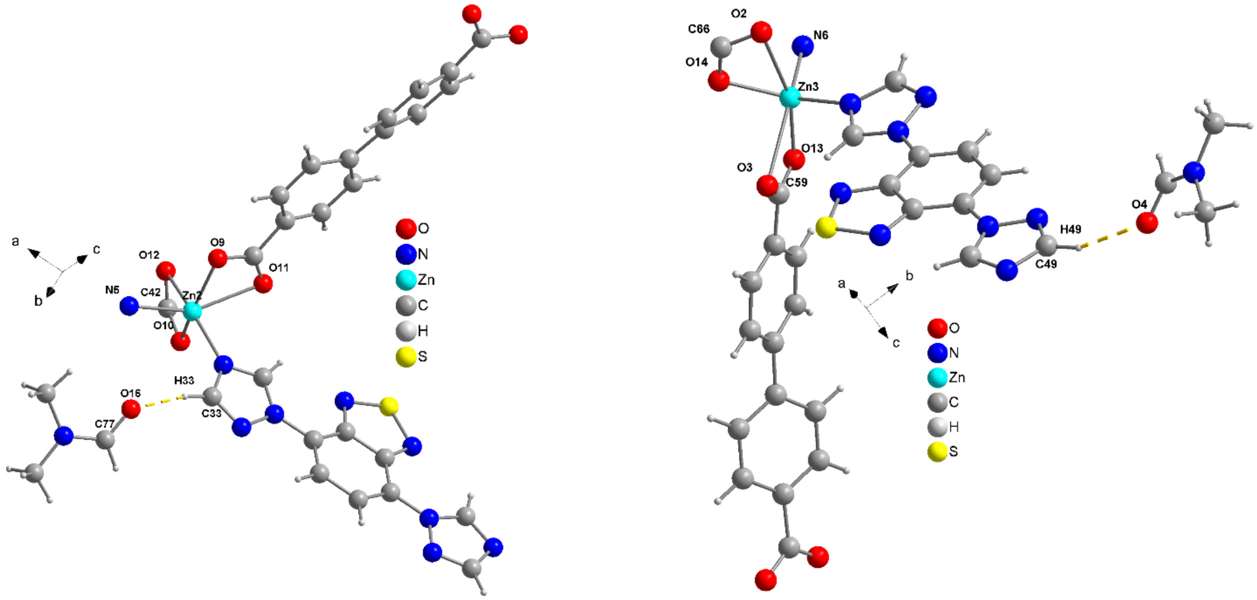

Figure S1. Hydrogen bonds between DMF molecules and 1,2,4-triazole fragments in the crystal structure of **1**.

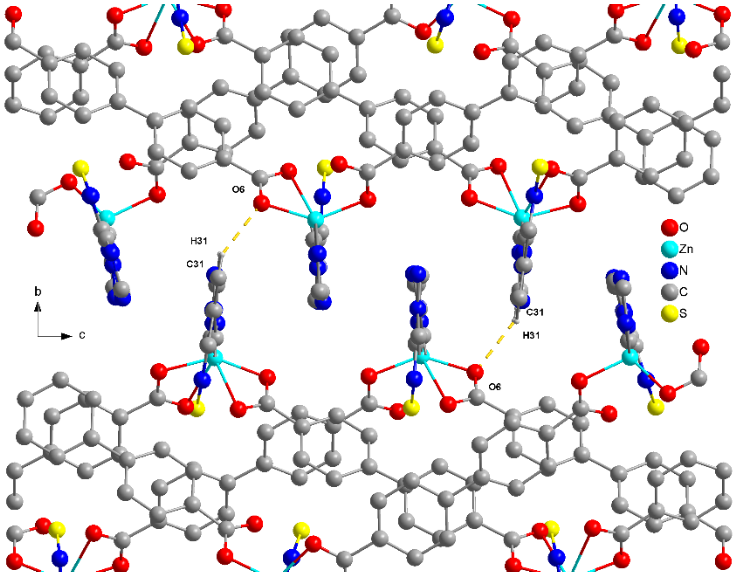

Figure S2. Interlayer hydrogen bonds in the crystal structure of **1**.

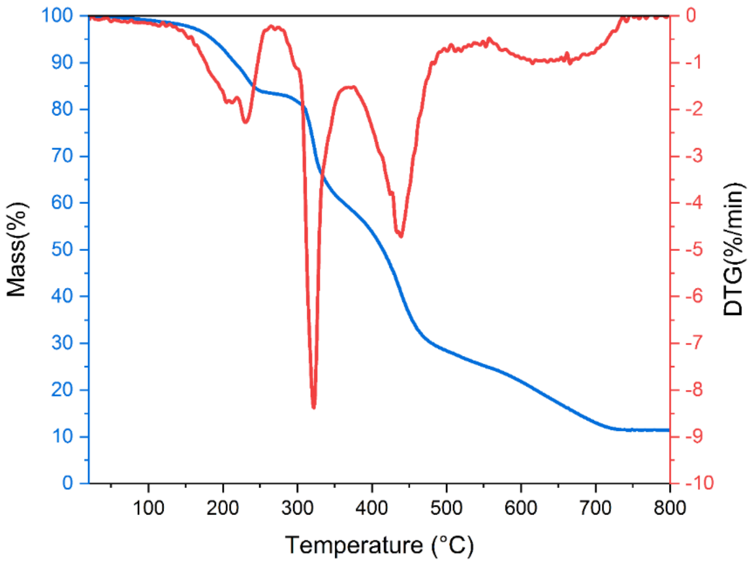

Figure S3. TGA curves of the coordination polymer **1**.

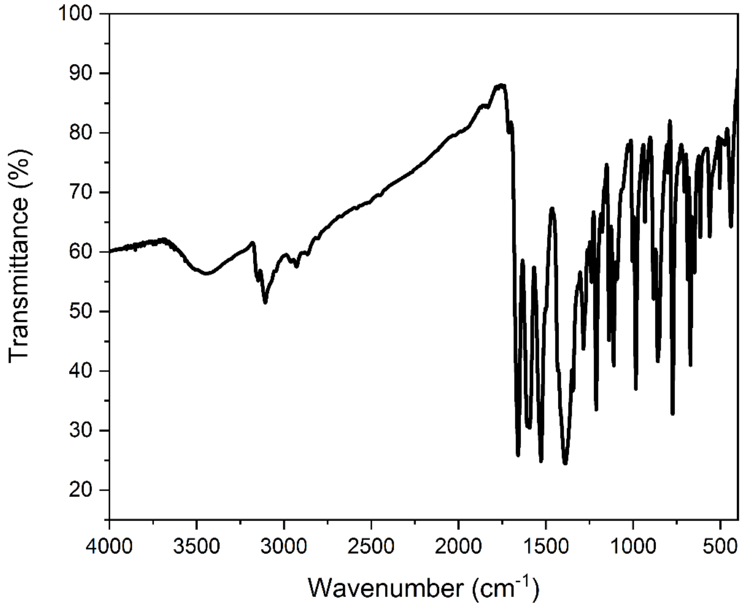

Figure S4. IR spectrum of the coordination polymer **1** in KBr pellet.

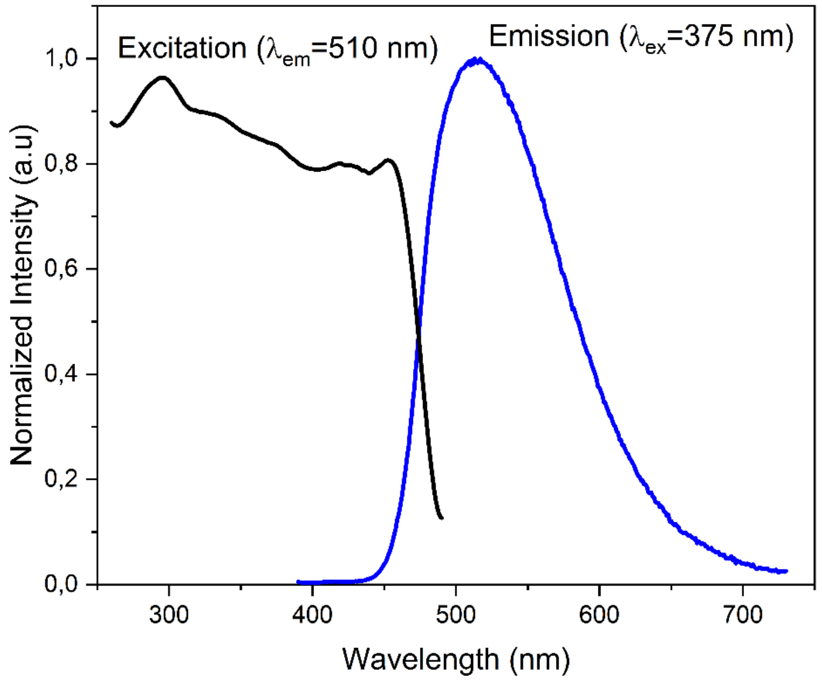

Figure S5. Excitation and emission spectra of the tr_2btd ligand.

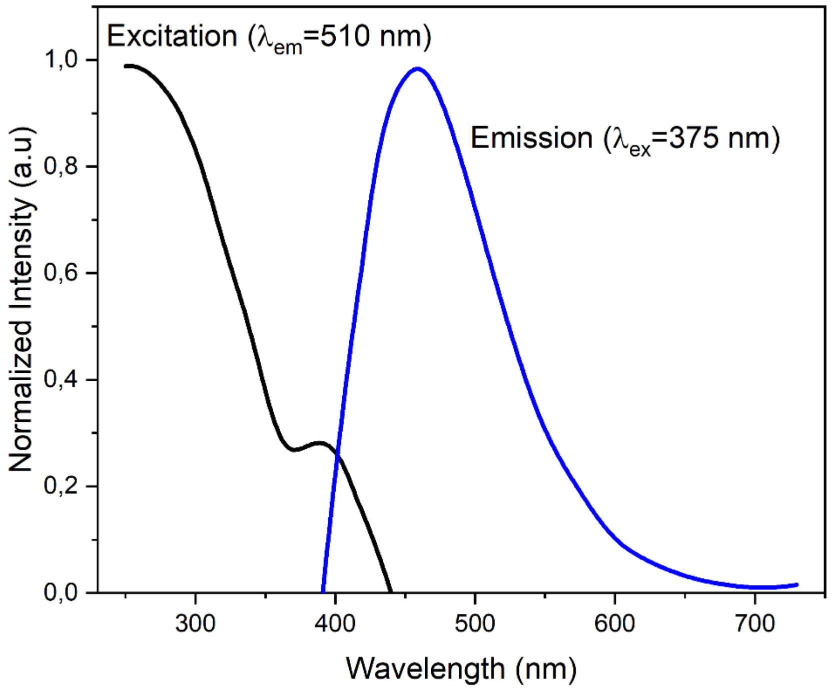

Figure S6 Excitation and emission spectra of the H_2bpdc ligand.

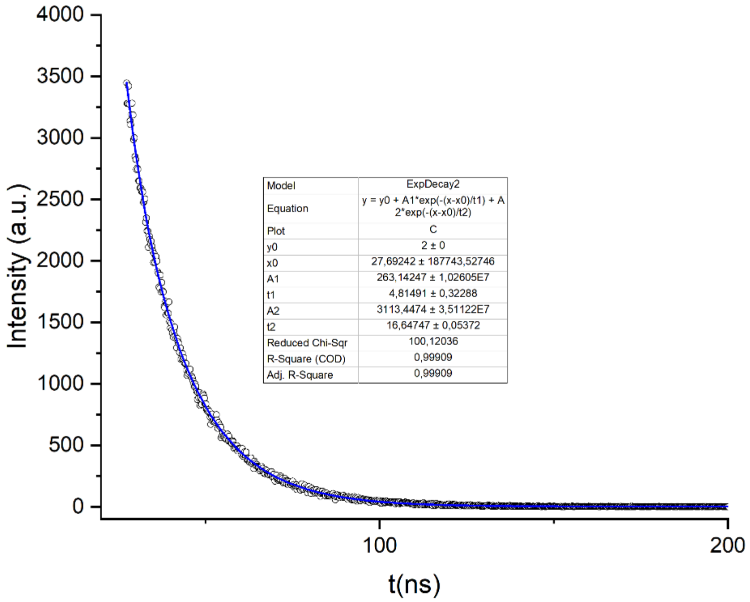

Figure S7. Fluorescence decay for **1** suspension in DMA.

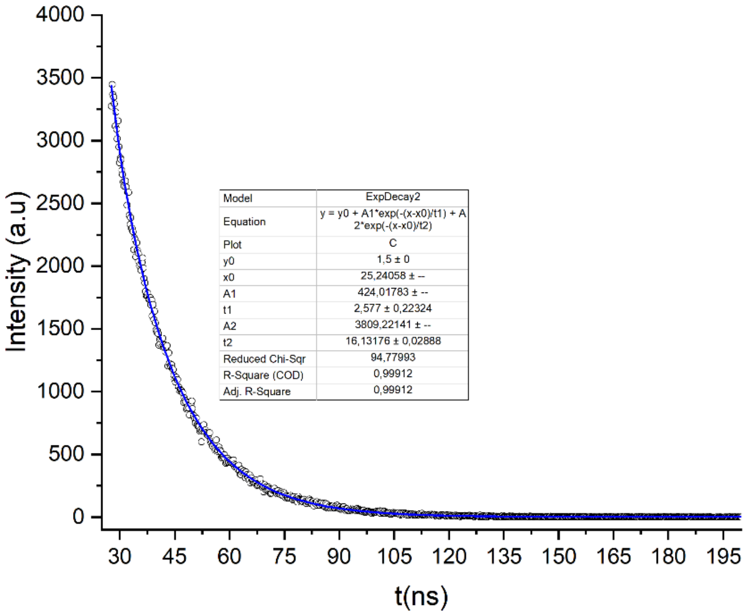

Figure S8. Fluoresce decay for **1** suspension in DMA in the presence of Al^{3+}.

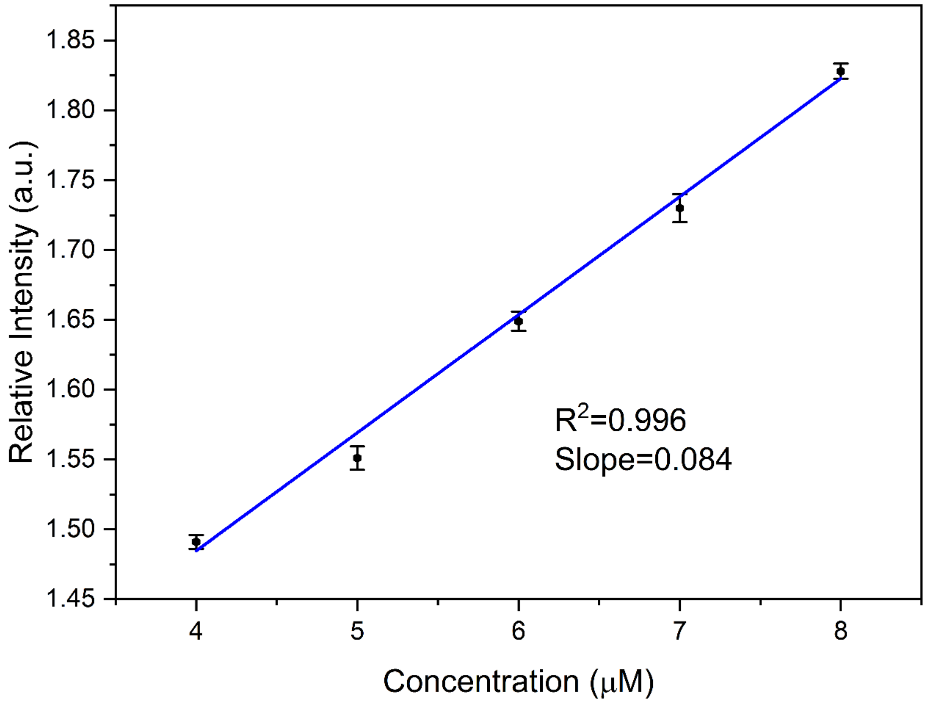

Figure S9. Linear fit for higher concentration range of intensity-concentration correlation.

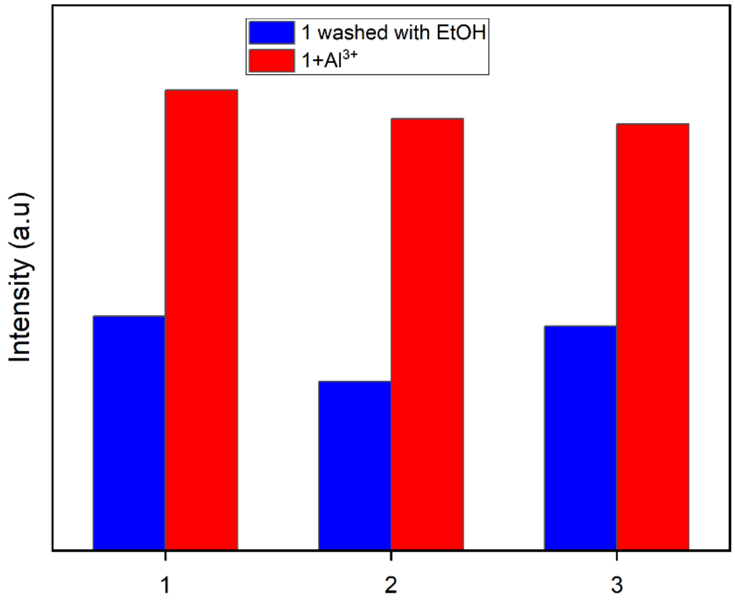

Figure S10. Fluorescence intensity of **1** in sensor recycling experiments.

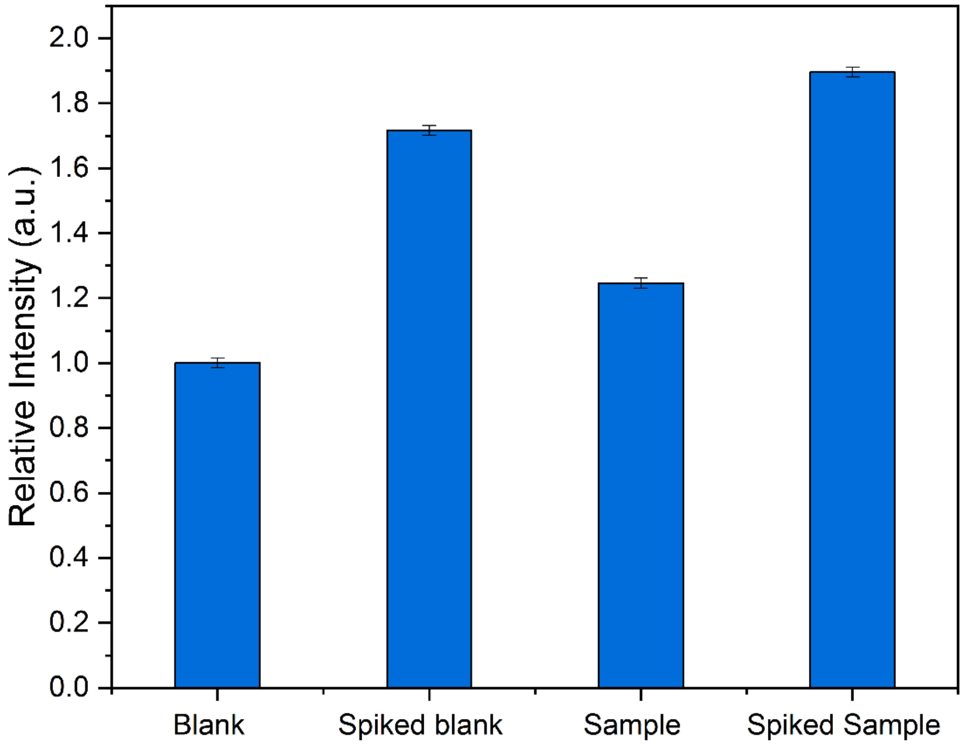

Figure S11. Fluorescence intensity of **1** in experiments with real sample and spiking.

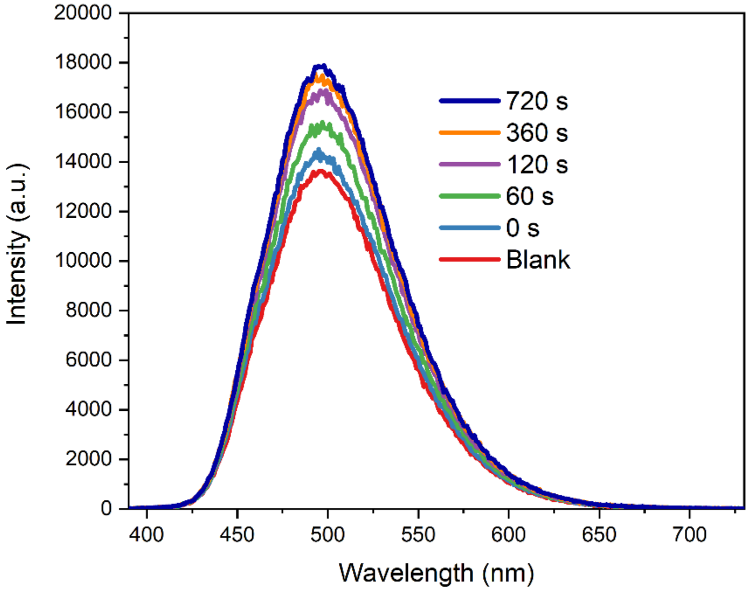

Figure S12. Time-dependent emission spectra of **1** DMA suspension upon addition of Al^{3+}.

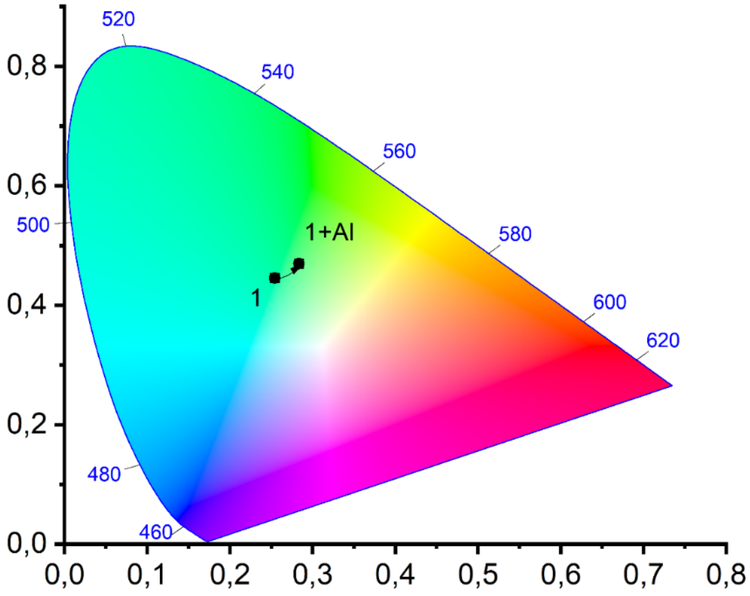

Figure S13. CIE 1931 chromaticity diagram with color coordinates for **1** and **1** + Al^{3+}.

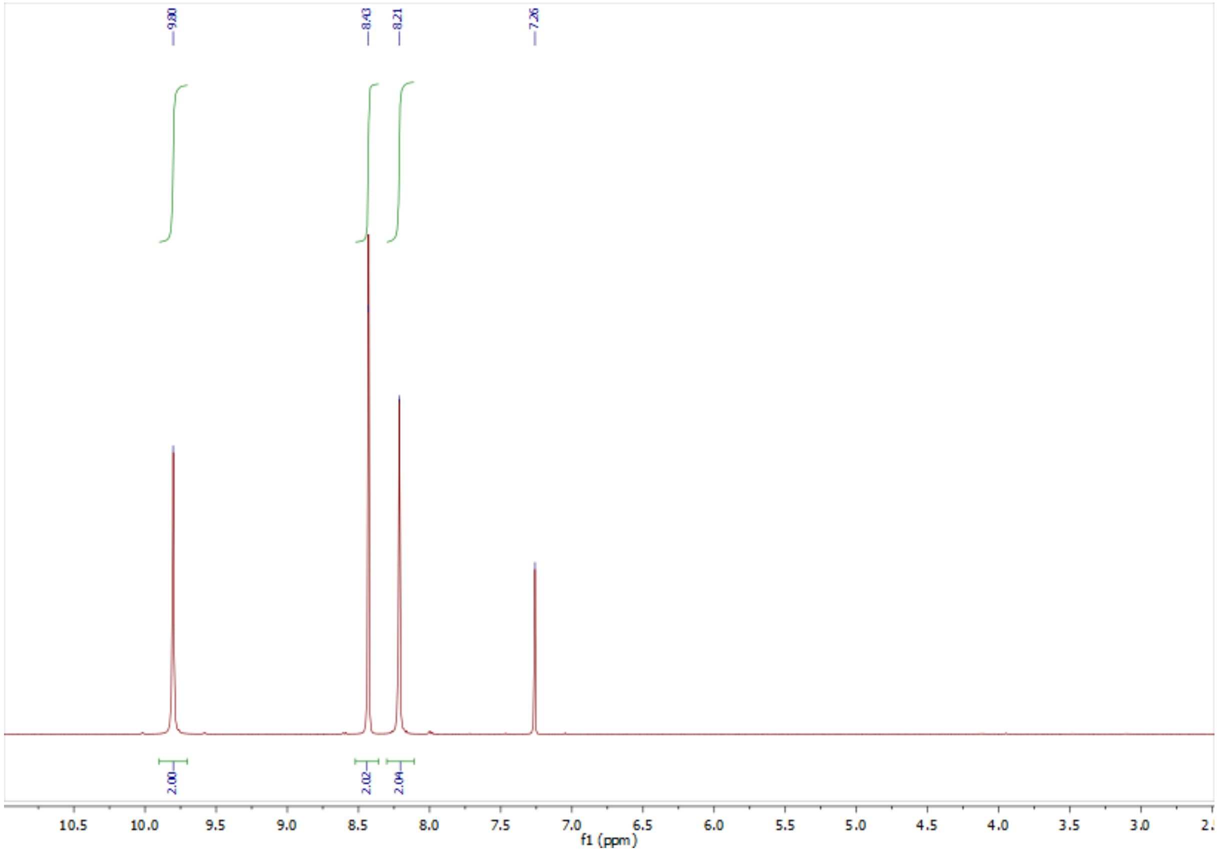

Figure S14. ^{1}H NMR spectra of tr_2btd ligand in $CDCl_3$ recorded at 500 MHz.

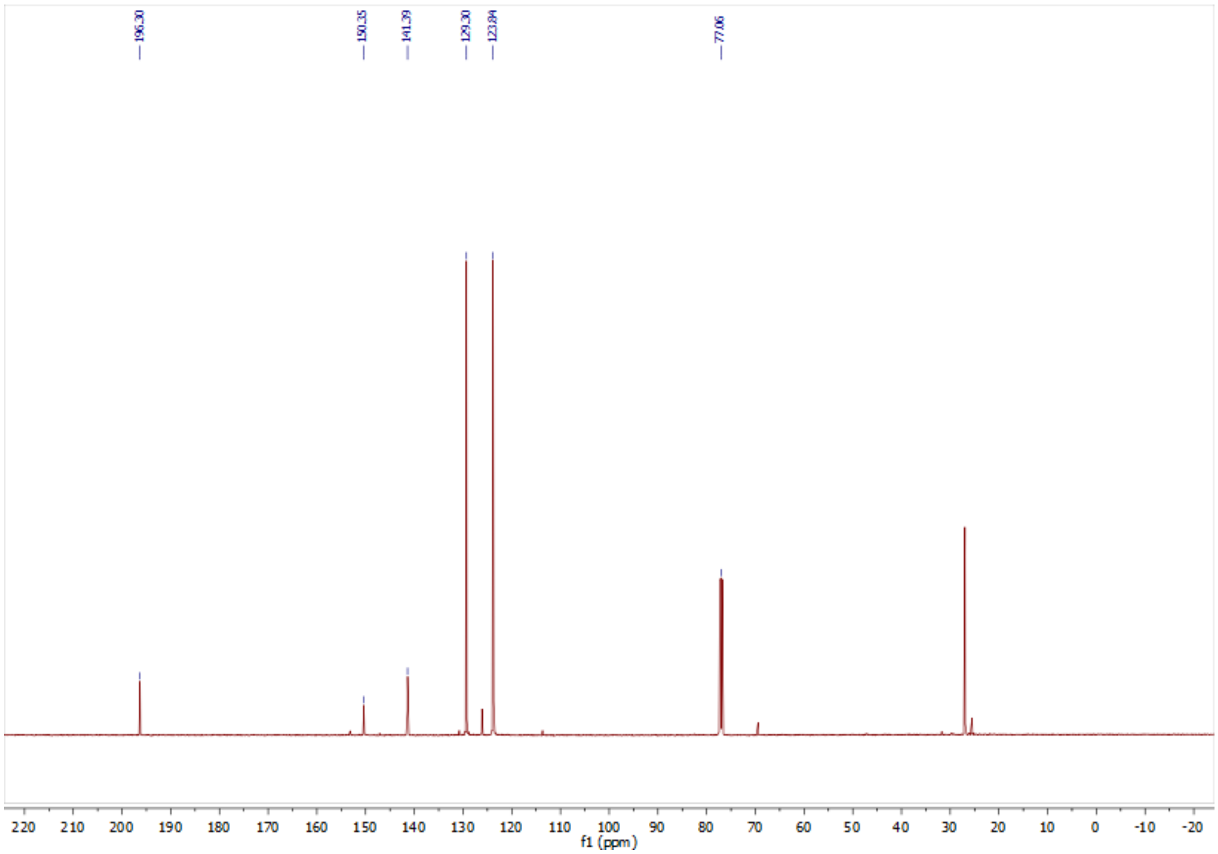

Figure S15. ^{13}C NMR spectra of tr_2btd ligand in $CDCl_3$ recorded at 125 MHz.
