# Supplementary material for: A Zn(II)-Based Sql Type 2D Coordination Polymer as a Highly Sensitive and Selective Turn-On Fluorescent Probe for Al3+
Source: Molecules. 2021 Dec 6;26(23):7392. doi: 10.3390/molecules26237392 (PMC8658932; doi:10.3390/molecules26237392)

checkCIF/PLATON report

Structure factors have been supplied for datablock(s) pvl632_a_sq

THIS REPORT IS FOR GUIDANCE ONLY. IF USED AS PART OF A REVIEW PROCEDURE FOR PUBLICATION, IT SHOULD NOT REPLACE THE EXPERTISE OF AN EXPERIENCED CRYSTALLOGRAPHIC REFEREE.

No syntax errors found. CIF dictionary Interpreting this report

Datablock: pvl632_a_sq

Bond precision: C-C = 0.0041 A Wavelength=0.71073

Cell:	a=13.3256(3)	b=25.4765(6)	c=25.8209(7)
	alpha=90	beta=104.918(1)	gamma=90
Temperature:	150 K		

	Calculated	Reported
Volume	8470.5(4)	8470.5(4)
Space group	P 21/c	P 21/c
Hall group	-P 2ybc	-P 2ybc
Moiety formula	C24 H14 N8 O4 S Zn, C3 H7 N O [+ solvent]	?
Sum formula	C27 H21 N9 O5 S Zn [+ solvent]	C27 H21 N9 O5 S Zn
Mr	648.98	648.98
Dx,g cm-3	1.527	1.526
Z	12	12
Mu (mm-1)	0.999	0.999
F000	3984.0	3979.0
F000'	3990.25	
h,k,lmax	19,36,36	19,36,36
Nref	25870	25841
Tmin,Tmax	0.887,0.932	0.652,0.742
Tmin'	0.771	

Correction method= # Reported T Limits: Tmin=0.652 Tmax=0.742
AbsCorr = MULTI-SCAN

Data completeness= 0.999 Theta(max)= 30.518

R(reflections)= 0.0563(17090) wR2(reflections)= 0.1417(25841)

S = 1.028 Npar= 1205

The following ALERTS were generated. Each ALERT has the format
test-name_ALERT_alert-type_alert-level.
Click on the hyperlinks for more details of the test.

Alert level C

```
PLAT213_ALERT_2_C Atom N2             has ADP max/min Ratio .....        3.8 prolat
PLAT213_ALERT_2_C Atom N3             has ADP max/min Ratio .....        4.0 prolat
PLAT213_ALERT_2_C Atom O2             has ADP max/min Ratio .....        3.2 prolat
PLAT215_ALERT_3_C Disordered S2'      has ADP max/min Ratio .....        3.2 Note
PLAT220_ALERT_2_C NonSolvent   Resd 1  N   Ueq(max)/Ueq(min) Range       4.1 Ratio
PLAT220_ALERT_2_C NonSolvent   Resd 3  O   Ueq(max)/Ueq(min) Range       3.8 Ratio
PLAT241_ALERT_2_C High   'MainMol' Ueq as Compared to Neighbors of        N2 Check
PLAT241_ALERT_2_C High   'MainMol' Ueq as Compared to Neighbors of        N3 Check
PLAT244_ALERT_4_C Low    'Solvent' Ueq as Compared to Neighbors of       N28 Check
PLAT244_ALERT_4_C Low    'Solvent' Ueq as Compared to Neighbors of        N1 Check
PLAT250_ALERT_2_C Large U3/U1 Ratio for Average U(i,j) Tensor ....       2.7 Note
PLAT250_ALERT_2_C Large U3/U1 Ratio for Average U(i,j) Tensor ....       3.4 Note
PLAT906_ALERT_3_C Large K Value in the Analysis of Variance ......     2.876 Check
PLAT911_ALERT_3_C Missing FCF Refl Between Thmin & STh/L=    0.600         5 Report
PLAT975_ALERT_2_C Check Calcd Resid. Dens.  0.99A   From O2             1.28 eA-3
PLAT975_ALERT_2_C Check Calcd Resid. Dens.  0.82A   From O14            0.54 eA-3
PLAT976_ALERT_2_C Check Calcd Resid. Dens.  0.84A   From O2            -0.51 eA-3
```

Alert level G

```
PLAT004_ALERT_5_G Polymeric Structure Found with Maximum Dimension         2 Info
PLAT083_ALERT_2_G SHELXL Second Parameter in WGHT  Unusually Large      9.94 Why ?
PLAT300_ALERT_4_G Atom Site Occupancy of S2         Constrained at       0.5 Check
PLAT300_ALERT_4_G Atom Site Occupancy of S2'        Constrained at       0.5 Check
PLAT300_ALERT_4_G Atom Site Occupancy of O17        Constrained at       0.6 Check
PLAT300_ALERT_4_G Atom Site Occupancy of O1         Constrained at       0.4 Check
PLAT300_ALERT_4_G Atom Site Occupancy of C81        Constrained at       0.6 Check
PLAT300_ALERT_4_G Atom Site Occupancy of C181       Constrained at       0.6 Check
PLAT300_ALERT_4_G Atom Site Occupancy of C82        Constrained at       0.4 Check
PLAT300_ALERT_4_G Atom Site Occupancy of C180       Constrained at       0.4 Check
PLAT300_ALERT_4_G Atom Site Occupancy of H81A       Constrained at       0.6 Check
PLAT300_ALERT_4_G Atom Site Occupancy of H81B       Constrained at       0.6 Check
PLAT300_ALERT_4_G Atom Site Occupancy of H81C       Constrained at       0.6 Check
PLAT300_ALERT_4_G Atom Site Occupancy of H181       Constrained at       0.6 Check
PLAT300_ALERT_4_G Atom Site Occupancy of H18A       Constrained at       0.4 Check
PLAT300_ALERT_4_G Atom Site Occupancy of H18B       Constrained at       0.4 Check
PLAT300_ALERT_4_G Atom Site Occupancy of H18C       Constrained at       0.4 Check
PLAT300_ALERT_4_G Atom Site Occupancy of H82        Constrained at       0.4 Check
PLAT301_ALERT_3_G Main Residue  Disorder .............(Resd  1  )         3% Note
PLAT302_ALERT_4_G Anion/Solvent/Minor-Residue Disorder (Resd  4  )       60% Note
PLAT380_ALERT_4_G Incorrectly? Oriented X(sp2)-Methyl Moiety .....       C76 Check
PLAT412_ALERT_2_G Short Intra XH3 .. XHn     H79A     ..H181     .      2.01 Ang.
                                                    x,y,z  =      1_555 Check
PLAT432_ALERT_2_G Short Inter X...Y Contact  S2'      ..C45             3.25 Ang.
                                              1-x,1-y,1-z  =      3_666 Check
```

```
PLAT432_ALERT_2_G Short Inter X...Y Contact  O1       ..C7              2.92 Ang.
                                                     x,y,z  =      1_555 Check
PLAT605_ALERT_4_G Largest Solvent Accessible VOID in the Structure       288 A**3
PLAT794_ALERT_5_G Tentative Bond Valency for Zn1       (II)      .       1.93 Info
PLAT794_ALERT_5_G Tentative Bond Valency for Zn2       (II)      .       1.91 Info
PLAT794_ALERT_5_G Tentative Bond Valency for Zn3       (II)      .       1.93 Info
PLAT869_ALERT_4_G ALERTS Related to the Use of SQUEEZE Suppressed          ! Info
PLAT883_ALERT_1_G No Info/Value for _atom_sites_solution_primary .     Please Do !
PLAT910_ALERT_3_G Missing # of FCF Reflection(s) Below Theta(Min).         1 Note
PLAT912_ALERT_4_G Missing # of FCF Reflections Above STh/L=  0.600        23 Note
PLAT933_ALERT_2_G Number of OMIT Records in Embedded .res File ...         4 Note
PLAT941_ALERT_3_G Average HKL Measurement Multiplicity ..........        4.1 Low
PLAT978_ALERT_2_G Number C-C Bonds with Positive Residual Density.         5 Info
```

---

   0 **ALERT level A** = Most likely a serious problem - resolve or explain
   0 **ALERT level B** = A potentially serious problem, consider carefully
  17 **ALERT level C** = Check. Ensure it is not caused by an omission or oversight
  35 **ALERT level G** = General information/check it is not something unexpected

   1 ALERT type 1 CIF construction/syntax error, inconsistent or missing data
  18 ALERT type 2 Indicator that the structure model may be wrong or deficient
   6 ALERT type 3 Indicator that the structure quality may be low
  23 ALERT type 4 Improvement, methodology, query or suggestion
   4 ALERT type 5 Informative message, check

It is advisable to attempt to resolve as many as possible of the alerts in all categories. Often the minor alerts point to easily fixed oversights, errors and omissions in your CIF or refinement strategy, so attention to these fine details can be worthwhile. In order to resolve some of the more serious problems it may be necessary to carry out additional measurements or structure refinements. However, the purpose of your study may justify the reported deviations and the more serious of these should normally be commented upon in the discussion or experimental section of a paper or in the "special_details" fields of the CIF. checkCIF was carefully designed to identify outliers and unusual parameters, but every test has its limitations and alerts that are not important in a particular case may appear. Conversely, the absence of alerts does not guarantee there are no aspects of the results needing attention. It is up to the individual to critically assess their own results and, if necessary, seek expert advice.

Publication of your CIF in IUCr journals

A basic structural check has been run on your CIF. These basic checks will be run on all CIFs submitted for publication in IUCr journals (*Acta Crystallographica*, *Journal of Applied Crystallography*, *Journal of Synchrotron Radiation*); however, if you intend to submit to *Acta Crystallographica Section C* or *E* or *IUCrData*, you should make sure that full publication checks are run on the final version of your CIF prior to submission.

Publication of your CIF in other journals

Please refer to the *Notes for Authors* of the relevant journal for any special instructions relating to CIF submission.

PLATON version of 13/07/2021; check.def file version of 13/07/2021

Datablock pvl632_a_sq - ellipsoid plot

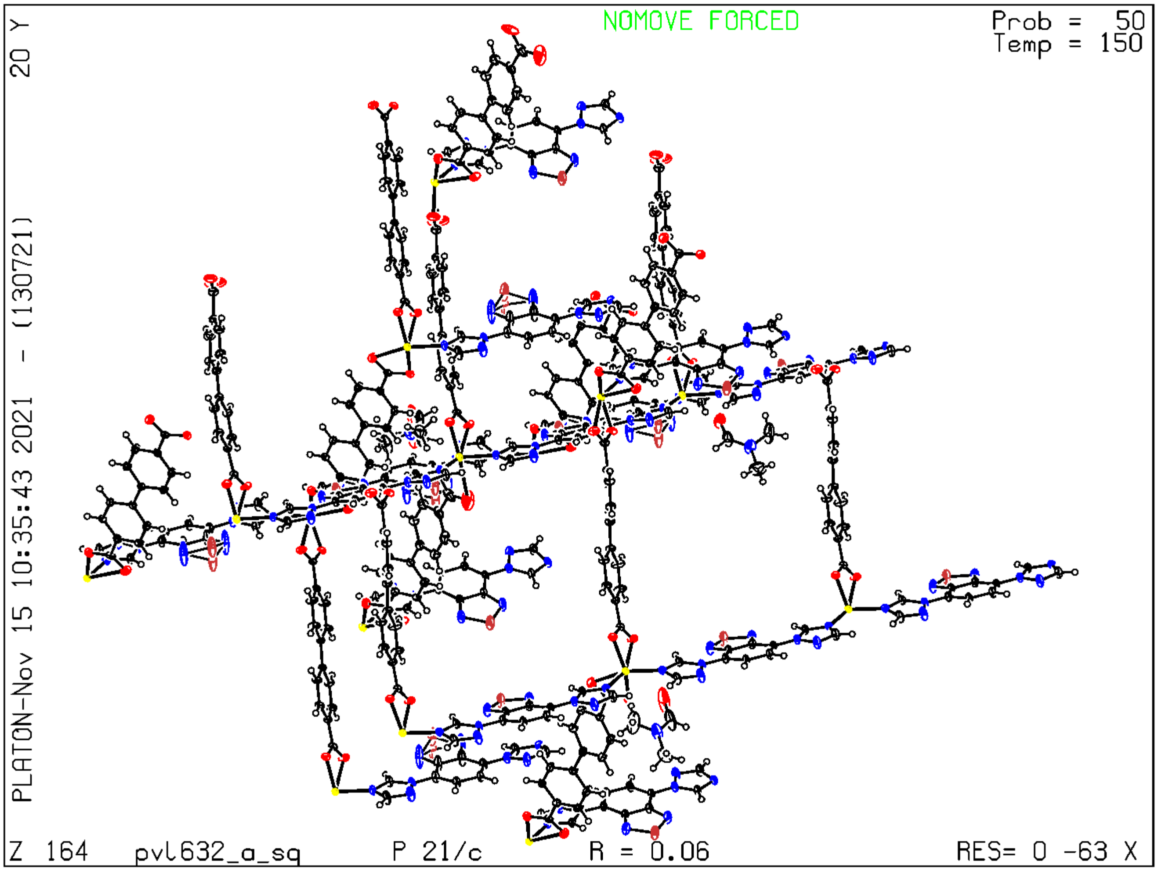

Supplement: Supplementary file 1 [file molecules-26-07392-s001.zip › checkcif.pdf]
